# What Works and What Does Not: A Discussion of Popular Approaches for the Abandonment of Female Genital Mutilation

**DOI:** 10.1155/2013/348248

**Published:** 2013-04-23

**Authors:** R. Elise B. Johansen, Nafissatou J. Diop, Glenn Laverack, Els Leye

**Affiliations:** ^1^Department of Reproductive Health and Research, World Health Organization, 20 Avenue Appia, 1211 Geneva 27, Switzerland; ^2^UNFPA-UNICEF Joint Programme on FGM/C, United Nations Population Fund, 304 East 42nd Street, New York, NY 10017, USA; ^3^Flinders University, Flinders Prevention, Promotion and Primary Health Care, Southgate Institute South Australia, GPO Box 2100, Adelaide, SA 5001, Australia; ^4^International Centre for Reproductive Health, De Pintelaan 185 UZP114, 9000 Ghent, Belgium

## Abstract

The prevalence of Female Genital Mutilation (FGM) is reducing in almost all countries in which it is a traditional practice. There are huge variations between countries and communities though, ranging from no change at all to countries and communities where the practice has been more than halved from one generation to the next. Various interventions implemented over the last 30–40 years are believed to have been instrumental in stimulating this reduction, even though in most cases the decrease in prevalence has been slow. This raises questions about the efficacy of interventions to eliminate FGM and an urgent need to channel the limited resources available, where it can make the most difference in the abandonment of FGM. This paper is intended to contribute to the design of more effective interventions by assessing existing knowledge of what works and what does not and discusses some of the most common approaches that have been evaluated: health risk approaches, conversion of excisers, training of health professionals as change agents, alternative rituals, community-led approaches, public statements, and legal measures.

## 1. Introduction

Over the past 30–40, years a range of interventions has been carried out to promote the abandonment of FGM with varied success [1]. Where the Demographic and Health Survey (DHS) and the Multiple Indicators Cluster Survey (MICS) data are available for example, a variation ranging from 16% in Kenya to 0% in the Gambia [2] has been observed. This paper discusses existing evidence to provide guidance on how best to design and implement programmes that promote the abandonment of FGM. Reviews and evaluations have been conducted to ascertain the effectiveness of programmes to eliminate FGM, and this suggests that some approaches are more successful than others. 

*Key Facts*. FGM is defined as any procedure that cuts or harms females' genitalia without medical indication [3]. In the 28 countries, from which prevalence data does exist, an estimated 101 million girls and women above 9 years have undergone FGM [4], and 3.3 million girls are at risk of being subjected to FGM annually. In these 28 countries the prevalence of FGM ranges from 0.6% to 98% although the practice of FGM is also found in other countries, including among migrants from FGM practicing countries [5]. 

Overall, the prevalence of FGM has declined, and in almost all countries girls and young women are less likely to have undergone FGM than older women. The pace of reduction varies widely, however, and millions of girls remain exposed to the risk of FGM in the future [4].

*Reflecting on Evidence from Common Approaches*. In this paper, we discuss existing evidence on the effectiveness of the most common approaches in light of our personal knowledge and experiences. Johansen, Diop, and Leye have worked on the issue of FGM for many years, designing and evaluating interventions and carrying out research. Laverack has experience of analysing community empowerment in a wide variety of health issues. Our discussions build on available reviews [6–12], of which only one is systematic [9]. We also use evaluations of individual interventions that fall into the categories discussed here.

*Successes and Challenges of Common FGM Approaches*. Our discussion of the most efficient approaches in ensuring the abandonment of FGM faces three major challenges. First is the limited extent to which interventions have been properly documented and evaluated. A systematic review from 2012 only found eight interventions that had been evaluated sufficient to be included in the review [9]. Secondly, many interventions combine two or more approaches and methods, and there is limited knowledge on the interplay and relative efficacy of the different components of an intervention. Thirdly, most interventions do not have the total abandonment of all forms of FGM as the objective, though this is mostly an ultimate goal. Considering what is feasible within the available timeframe and budget, most interventions aim at secondary outcomes. This can be to break the silence and to initiate critical reflection upon FGM. Others aim at increasing knowledge and awareness of its association with health complications and its violation of human rights. Others again aim to change attitudes and intentions with regards to FGM [9] or at modifying the practice, either through reducing the extent of cutting [13], promoting its medicalization (e.g., in Egypt and Indonesia) [14] or changing the age at which FGM is carried out (e.g., in Sierra Leone). 

However, while these secondary targets are considered as a first step towards total abandonment, and many see it as a stage of change, evidence shows that the translation of these secondary goals into actual abandonment of FGM is far from automatic. There are, for example, many surveys that find women who express a negative attitude to the continuation of FGM, while they still intend to let their daughters undergo the practice.

A major reason for this apparent contradiction between attitude and behaviour is a social and cultural pressure to uphold the tradition. Therefore, the importance of a community-wide change to enable individual families to abandon FGM is now widely recognized [3, 15]. Experience shows that large-scale abandonment can only be expected when FGM is no longer an all-dominant social norm and families can abandon the practice without the risk of stigmatization and exclusion. 

We will now discuss seven of the most common approaches that have undergone some form of evaluation: (1) health risk approaches, (2) conversion of excisers, (3) training of health professionals as change agents, (4) alternative rituals, (5) community-led approaches, (6) public statements, and (7) legal measures.

Table 1 provides asummary of the main advantages and challenges of popular approaches towards the abandonment of FGM.

## 2. Health Risk Approaches

Since interventions against FGM first started more than 40 years ago, providing information about the health risks associated with FGM has been the most popular approach. It builds on the idea that if people are informed about the negative health effects of FGM, they will abandon the practice. Health risk interventions have been targeted at various population groups both as a stand-alone activity and as part of other interventions [11, 12]. In its crudest form, it can include delivering factual and didactic messages around the physical complications of FGM by local health providers, community facilitators, or NGO staff [7]. In its broadest form, it includes local knowledge and personal sharing and reflection coupled with the provision of health care services for complications of FGM [16].

It is believed that an increased knowledge of the negative health effects can stimulate reflection and critical thinking, leading to reduce the approval of, and eventually to the abandonment of, FGM. 

Evidence suggests that the negative health effects of FGM presented by a health authority such as a medical professional are a key motivational factor for religious leaders to take a clear and strong stance against the practice, and which might lead to the issue religious edicts (Fatwa) against FGM [17]. Such Fatwas were issued, for example, in Egypt in 2006 [18], in Mauritania in 2010 [19], followed by a West African subregional fatwa in 2012 [17]. Fatwa's are believed to contribute to change in communities where people link the practice of FGM to Islam. 

Health information has also influenced policy makers to promote laws and regulations such as the care for complications in Mali [20] and recently adopted legislation in Kenya and Guinea Bissau. Media attention given to health complications can also have positive effects. In 2007, the death of two girls after FGM was carried out by health care providers in Egypt was instrumental in strengthening legislation against the practice. The realization, publication, and spreading of the results of clinical studies on the negative health effects of FGM can lead to positive changes in terms of engagement of national authorities. A clinical study in 2009 and requested by the Gambian Vice-President and Minister of Women's Affairs demonstrated for the first time the magnitude of immediate and long-term health consequences of FGM in the country [21]. The results of this study were a key instrument to ensure institutional pre- and in-service training for all health personnel.

There are however also challenges to health risk approaches.

### 2.1. Medicalization or Change of Type to Reduce the Health Risks

Experience suggests that health information can lead to changes other than abandonment, most commonly an increase in the extent to which health providers are performing FGM [14, 20, 22], a trend associated with a risk of institutionalisation and continuation of FGM rather than its abandonment. It can also lead to the intention to change the type of FGM, as noted among others in Somalia and Sudan [23, 24]. 

### 2.2. People May Not Believe the Information Given

One study in the Gambia and Senegal showed that only those who were already critical to FGM believed in the information of health risks [25, 26]. One reason for this is that the immediate complications of FGM are often attributed to other factors such as witchcraft or evil spirits [27, 28]. Another aspect is a gap between the information given and people's personal experiences, because not all women experience health complications and those who do tend to keep silent about it. Finally, both women and health professionals have generally been found not to attribute long-term health consequences to FGM [16, 21, 26, 29]. 

### 2.3. Inadequate Quality of the Information

Several researchers have pointed to the difficulties posed by employing a “laundry list” of health risks that is not adapted to the local setting [30]. For example, though only approximately 10% of all FGM cases are infibulations (Type III FGM), the “health risk approach” often highlights complications mainly associated with type III as common complications of all types of FGM, whilst other common complications (e.g., cysts and scarring) are rarely mentioned.

### 2.4. Condemning, Violent, and Shocking Messaging Can Provoke Defence Reactions

To highlight the negative health effects of FGM, health information messages often use strong visual images, such as the use of razor-blades and blood. However, one review found that such messages were seen as imposing (e.g., “Stop excising”), demoralising (e.g., “Stop excision or your daughter will die”), or were difficult for people to understand [8]. 

### 2.5. Health Risks Are Considered a Lesser Danger Than the Dangers Associated with Abandoning FGM 

In communities where FGM is common, it is upheld as a social norm and enforced through social sanctions of individuals or families that do not conform. The risk of being socially ostracized, excluded from community activities, denied financial and practical support, as well as marriage possibilities, can outweigh the health risks associated with the practice [26, 29].

## 3. Conversion of Excisers

The vast majority of FGM in Africa, around 80%, is carried out by traditional practitioners, that is, excisers [31]. A popular approach has been to target excisers to convince them to stop performing FGM. Such interventions usually include education on the physiology of female genitalia, the harmful health consequences of FGM, their role in perpetuating it, and encouragement to stop performing FGM. In some cases, training and financial support is provided for excisers to help them find sources of income other than performing FGM [32, 33]. The expected outcome is a reduction in the numbers of excisers performing FGM subsequently leading to a reduction in the number of FGM performed. 

One advantage of this type of intervention is their clear and limited scope and consequently clear and simple indicators to measure success, that is, number of excisers “dropping their knife” [27]. Furthermore, reports of public ceremonies of “dropping knives” shown in the media provide visibility to the issue of FGM and can stimulate debate. However, several concerns have been observed with the conversion of excisers. 

### 3.1. Converted Excisers May Continue to Practice or Hand over Their Knife to Apprentices

A major review found that many excisers did not keep their promise to stop performing FGM [8]. In Mali, an evaluation found that 29 of the 41 excisers interviewed after completing the conversion programme declared that they still performed FGM and were not convinced that what they were doing was wrong. Also, most people in the study sites did not know of any practitioners who had stopped working as excisers [32]. 

Furthermore, if there is no change in the request for FGM, other persons will step in to fill the demand, including other excisers, health care providers, or newcomers [34]. For example, in Kenya, excisers stopped because their services had been taken over by health providers [29]. In some cases, excisers handed over their post to their apprentices, who are often family members [34], while in other settings excisers are brought in from other regions or countries [26]. 

### 3.2. Ex-Excisers May Not Be Considered Reliable When Turning against FGM

A key motivation for converting excisers is to take advantage of, and uphold, the respected position they are alleged to have in the community. However, few assessments of their social role and position are reported. Ethnographic research and the experience of the authors of this paper in the field display a wide variety in the role of excisers from powerful ritual specialists (e.g., in Liberia and Sierra Leone) to low cast or stigmatized ethnic groups (e.g., in Senegal, Mali, and Somalia) [23, 27]. Furthermore, being an exciser is rarely a full-time engagement and is usually combined with other tasks, such as support in childbirth. Little is known about the extent to which community members are listening to or are convinced by the arguments of converted excisers [27]. 

### 3.3. Income Might Not Be a Major Motivation for Excisers

Alternative income for excisers is meant to compensate for their loss in income if they give up FGM. However, existing information indicates that the financial gain for excisers is usually quite small (e.g., tokens of soap or food). Most excisers' duties are requested irregularly and hence the income from FGM appears to be a supplement rather than their main income [27]. Income might not be a major motive, and excisers in Mali, for example, felt that the funds received for dropping their knives' could not compensate for the social status associated with performing FGM [34].

Excisers share their often precarious living situations with the majority of the community. Some evidence suggests that singling out excisers for financial support and training in such precarious settings could contribute to internal conflicts and can boost the role of the excisers in the community or contribute to the recruitment of new excisers [35]. 

## 4. Training of Health Professionals as Change Agents

Several interventions have targeted health professionals, with the aim of preventing them from performing FGM, building their capacities to identify and treat complications and recruiting them as change agents [9, 35, 36]. 

Evaluations performed at the end of trainings for health professionals report an increased knowledge about FGM, health complications of FGM, and how to manage the complications, as well as an increased negative attitude to FGM [32, 36–38]. In an intervention study in the Gambia, health providers expressed shock, surprise, and anger when realising that the gynaecological complications they had been treating were consequences of FGM. This realisation contributed to a change in attitude and a willingness to engage in community outreach to prevent the practice [38].

However, interventions of engaging health providers as change agents have been met with a number of challenges.

### 4.1. Health Care Providers May Resist Working against FGM

Health care providers are often part of the same communities that support FGM. Hence, these health professionals may support FGM or be scared of involving themselves in such a sensitive issue [37, 39–41]. Studies have documented that some health care providers support FGM in general [21] and in its medicalized form in particular [22, 37, 40]. 

### 4.2. Content of the Training May Be Inadequate

The training time and content in the interventions varies widely from two 60-minute sessions [42] to five sessions of seven days with a comprehensive reproductive and child-health training [12]. Though no analysis comparing the content of the training is available, it is evident that shorter training gives less room for content, reflection, and discussion.

### 4.3. Systemic Difficulties in Putting Knowledge into Practice

The work burden and lack of inclusion of FGM relevant measures within standard procedures can inhibit a practical implementation of counselling and the provision of information [43]. The ability of health professionals to translate training into action both requires structural support, that is, in the form of resources and time allocated, and techniques, encouragement and empowerment strategies. A study from Mali [32] found that health care providers were not counselling against FGM after their training because of the extra burden on an already heavy workload. The effectiveness of the training will therefore depend on the position of health providers against FGM, their motivations and commitment to stand up against FGM, as well as the resources (including time) they have at their disposal to work on the topic.

## 5. Alternative Rites Programmes 

In many communities, FGM is part of a larger rite of passage, often around puberty, that facilitates and marks the integration of a girl as a more mature member of the community. In some of these communities, interventions have been developed to replace the rite of passage with FGM, by an alternative rite without FGM. Such alternative rites programmes are expected to fulfil the cultural tradition of a coming of age ritual, so that girls can be socially accepted without having to go through FGM. These interventions are believed to show positive attitude and respect for cultural traditions and thereby prevent defensive reactions against efforts to abandon FGM and to facilitate abandonment of FGM by maintaining the ritual framework [29, 44]. In other situations, a key motivating factor to implement such interventions has been to safeguard girls during ritual seasons [28]. In some cases, excising rituals are replaced with a version without FGM (e.g., in Sierra Leone and Kenya), in others, previously used rituals have been revitalised [44], or a new form of education and “marking” has been introduced [45]. 

In Kenya, such interventions were first developed by community-based organisations in consultation with community members, such as families and local political, ritual, and religious leaders [28, 44–46]. Commonly they consist of a period of training, often in seclusion, and a public celebration and/or a certificate to mark the conclusion of the ritual. The training itself often aims at empowering the young girls to take charge of their sexual and reproductive health and rights, and the ceremony functions as a public statement of abandonment of FGM. 

Two assessments in Kenya found that after this type of an intervention, more girls knew about reproductive health issues and expressed gender egalitarian attitudes, and more of the girls' families stated that there were no benefits to FGM and had increased knowledge about health, social and psychological problems associated with FGM. In addition, fewer people stated the intention to subject their daughters to FGM [45]. The promising aspects of this approach as it was first developed are the involvement of family and community members in designing the project and the entry point it can provide to promote dialogue among family members [28, 29, 35]. 

Some of the factors that can reduce the efficacy of such interventions however are the following.

### 5.1. Limited Integration of the Whole Community

There is a huge variation as to the extent in which families, and communities are involved in alternative rites programmes. Most interventions do include both girls and their families, but the extent to which the wider community is actively involved varies significantly. Alternative rites cannot be introduced without a preceding or accompanying process of sensitization in which an attitudinal change has to have occurred [45].

### 5.2. Insufficient Adaptation into the Specific Sociocultural Situation of Each Community

The role and meaning of traditional rites of passage and of FGM vary considerably between ethnic groups. For example, in some West African communities, the actual FGM and the confirming initiation ritual are often separated in time (e.g., in Senegal [47, 47], Sierra Leone [48, 48], and the Gambia [44]. In one programme in the Gambia, the girls that participated in the alternative ritual had already undergone FGM at an earlier age, and the purpose of the ritual was therefore to train the girls so that they would resist FGM on their future daughters [44]. 

## 6. Community-Led Approaches

Community-led programmes have been identified as a necessary factor to tackle the social convention of FGM [15, 35, 49]. Evaluations of FGM abandonment interventions suggest that community involvement is key to create sustainable change [28, 35]. Community-led interventions to abandon FGM aim at promoting the empowerment of women and girls and the community at large to enable them to critically examine their own tradition and to gain the power to abandon FGM for their own benefit [15, 29, 35, 45]. Empowerment refers to the process by which the girls, women, and their communities gain control over the factors and decisions that shape their lives [50]. Understood as an empowerment exercise, interventions usually integrate the issue into a wider learning package, including aspects such as gender and development, as well as the social, political, legal, health, and economic development of a community.

The current most widespread and systematically implemented community-led programmes show promising results [29, 51]. One of these consists of an education programme with four modules, covering hygiene, problem solving, women's health, and human rights and has been carried out in several countries [2]. Such programs may have positively affected the prevalence of FGM and participants' knowledge about the consequences of FGM [29, 51].

### 6.1. Success Varies between Communities

A long-term evaluation of an intervention in Senegal showed a reduction of prevalence in the youngest generation (0–9 years of age) of almost 70% compared to 40% in a control village. In another area in the same country the reduction was about 24% [10]. However, when run in neighboring Burkina Faso only 3% reduction was identified compared to the control group [51]. When the same programme was run in Somalia, the public declaration achieved was only to change the type of cutting, rather than abandonment of FGM [52]. Egypt had a similar experience, in that the success of a community-led programme in one village was not paralleled in the neighboring village, suggesting differences in social and religious factors as key to variation [25].

### 6.2. Insufficient Community-Engagement

*A particular* challenge with community-led approaches is that some interventions that claim to be empowering use vertical programs that “lecture” and “educate” the communities, rather than using a participatory approach to involve others to empower themselves [51].

## 7. Public Statements

An important element in the process of mobilizing communities is a public statement (often referred to as public declarations) of a decision to abandon FGM by a larger group, usually a significant part of a community. Such public statements both express and facilitate change in the social conventions of the community. Public statements can take different forms, including signing a statement, alternative rites of passage celebrations, and multivillage gatherings. 

A public statement can create a sense of collective change, which can help to empower families to abandon FGM and encourage others to follow. When public statements are made, this suggests that a sufficient number of individuals have decided to abandon FGM, which can further promote broad-scale abandonment. It is important to note however, that when a public statement has been made, this does not necessarily indicate that the whole community supports the abandonment of FGM and some may continue to do so. Depending on the stage of readiness for change and processes running prior to the public statements, they can mark a final decision already made to abandon FGM in some communities, whereas in others they are a milestone that signifies readiness for change, and further support is needed to sustain and accelerate the process [12]. 

Though public statements seem vital to facilitate large-scale change in high prevalence communities, there are certain risks.

### 7.1. Public Statements by Subgroups Only

Interventions ensuring public statements from subgroups rather than whole communities rarely result in abandonment, even when the selected subgroups form an authoritative voice, such as excisers, religious leaders, or men. For example, while public statements from men are expected to be influential due to their powerful role in society, evidence suggests that their potential influence is mitigated by the fact that the responsibility for FGM most often lies with the women [53]. Furthermore, while fatwas from high-ranking religious organizations or personalities are hoped to create change, the effect has never been systematically measured [54].

## 8. Legal Measures

Studies indicate that legislation and its implementation can have a preventive effect [26, 55, 56]. Most African countries with documented FGM have now passed laws against the practice. This provides an official legal platform for action and offers legal protection for women and can discourage excisers and families for fear of prosecution [35]. It can also offer health professionals a legal framework to oppose requests for performing FGM. 

Laws against FGM are an important policy commitment and create an enabling environment. When preceded and complemented by education campaigns and advocacy and the sensitization of leaders, as well as adequate implementation, their effect is expected to be higher. For example, it was found that the beginning of the abandonment of FGM in Burkina Faso mostly coincides with the time of the adoption and application of the law banning the practice. [57]. 

### 8.1. Challenges with Legislative Measures

One challenge to the effectiveness of legal measures is that the practice may go underground. FGM rituals appear to have diminished but instead the cutting has continued in some countries outside the law as a way to avoid legal implications [47]. In several contexts, laws and debates about passing or enforcing legal measures led to resistance and protest, as for example, in Senegal, Mali, and Egypt [17]. A final concern has been that the existence of a law may also scare people with immediate health complications after FGM from seeking health care [8]. 

## 9. Discussion

The rather slow decline in prevalence after nearly four decades of campaigning against FGM raises questions about the effectiveness of interventions to eliminate this practice. Some of the most commonly used approaches discussed in this paper have a notable lack of thorough evaluations. However, the existing information is sufficient to discuss some important successes and challenges. 

Evidence on the effectiveness of interventions is insufficient, particularly whether they have led to an actual decline in the incidence or prevalence of FGM. There is also insufficient evidence on whether secondary goals, such as change of attitude and increased knowledge, eventually lead to a decline in the practice. There is limited evidence on the key factors of successful interventions, especially since almost identical interventions can have very different results in different settings. Such information would be key to improve and scale up successful approaches. 

Interventions tend first to appeal to those that are already questioning or have abandoned the practice, seeing the intervention as a way to get social acceptance for their change [26, 29, 45]. This is a common pattern in social change [58] and helps those that are already converted to translate their conviction into action and gives a push towards change to those that are ambivalent. This seems to be a necessary first step that subsequently facilitates change among more conservative members of the community [58]. However, this might imply that the next steps trying to bring along the more sceptical or conservative groups may require a revision of approaches and support for a longer period.

The need for interventions to be driven by and involve the whole community underlines the importance of gaining in-depth knowledge of the community and the need to pay careful attention to strategies to engage with communities. Reviews have shown that targeting FGM is most effective and well received when a broader approach is used, assisting the community with other challenges [10, 51]. While conversion of leading persons such as community and religious leaders to speak out against FGM and a favourable legal framework create an enabling environment; evidence shows the importance of peer groups. Only when information comes from someone similar to oneself, are the majority of people willing to accept and adapt to the information [29, 51, 58].

Resources for the abandonment of FGM including financial support from foreign donors can be instrumental to get interventions carried out, and incentives can be key to ensure participation and engagement. However, it can also complicate the process. People who engage in interventions can be motivated by access to resources, such as money or power. Incentives can be used directly, such as sponsored weddings or education for uncut girls, alternative income for excisers, and compensation for taking part in training and meetings. Free training, funding of activities, and access to employment are more indirect incentives. If this leads to actual change in attitude and practice, the project can be seen as successful, though sustainability may be a challenge. But there is also a risk that the conviction is not genuine, and people just pretend to give support for the cause to get access to the resources promised. More common is an expressed mistrust of how genuine the message of leaders against FGM is, and people and their messages, have been accused of being “bought” by the donors. These issues are however rarely discussed in evaluation reports. 

We believe that many of the challenges discussed here can be overcome if they are included in a more comprehensive programme, rather than as stand-alone approach to abandon FGM.

Although information about health complications might be insufficient in bringing about large-scale changes alone, it is a key component in all interventions. People have a right to get information about health and the health complications associated with FGM. Concern over the health consequences of FGM is one of the most significant motivations for abandonment of the practice [9]. However, to be effective, it has to be reliable and communicated in a way that it can be absorbed and integrated into a wider health information package.

The involvement of health providers in identifying and caring for complications as well as disseminating their local knowledge might be a potential way to improve this approach. Training of health providers on all aspects of FGM needs to take into account that most health providers tend to share the same support for the practice as the community they serve. Therefore, interventions must ensure that they also take a stand against the practice and realise that medicalizing the practice will not benefit the community. The training must become integrated as a standard curriculum for all health providers, monitoring, and followup of trained providers being an essential part. 

Whereas conversion of excisers as a stand-alone activity cannot be expected to have an effect on the prevalence of FGM, including excisers in a comprehensive programmes can prevent a risk that they obstruct the intervention and that they are not ostracized from the community. Alternative rites programmes can be an effective approach only in communities in which FGM is a traditional part of such a ritual, and where girls, their families, and the whole community are involved in the efforts to abandon FGM. Interventions based on community engagement require long-term investment, a comprehensive education package, and a supportive context. It is key that it is well adapted to the local setting, including sociocultural and religious factors, as well as human resources (i.e., well-trained individuals). 

Sustainability is best achieved through the effect of change, in which the abandonment of FGM by a smaller group or community is disseminated to the larger community, for which organizational support is provided. Public statements can be an important way of making publicly known that the local social convention is changing. Such statements should be the result of a community-wide process to be effective, as statements made by subgroups have limited effect. Further research to investigate the effects of religiously founded public statements, such as fatwas against FGM, could be useful, and having legislation and policies in place provides support to people ready to change. 

## 10. Conclusion

Besides the above-mentioned importance of a comprehensive and holistic programme for the abandonment of FGM including different types of activities, the authors believe that it is equally important to have a thorough design and planning and local adaptation of the intervention. The basic content of this should be a situation analysis and baseline assessment prior to any intervention. This will establish a community's readiness to change, as well as other factors related to FGM (decision making processes, power dynamics, meanings attributed to FGM, etc). A good design should equally include plans and procedures for monitoring the process. This entails a thorough documentation of each step of the intervention. Finally, a good design should include evaluation of the output and outcomes to either compare the situation before and after and/or establish a comparison site. It should be taken into account that interventions require substantial time before they can result in actual change. The time needed may vary considerably between communities, depending on various local and contextual factors.

## Figures and Tables

**Table 1:** A summary of the main advantages and challenges of popular approaches towards the abandonment of FGM.

Approach	Advantages and potential successful results	Risks and disadvantages	Measures to overcome risks and disadvantages
Health risk info	(i) Stimulate resistance to FGM among lay people → reflection/abandonment religious leaders → fatwa politicians → laws and policies health providers → share information + denounce medicalization (ii) Improve health care for complications	(i) Medicalization (ii) Change type of FGM (ii) Disbelief (iv) Inadequate quality of information (v) Defence reactions (vi) Social norm overrules health risks	(i) Ensure health information is locally adapted, communicated nondjudgementally by a reliable source and combined with care for complications gibing space for reflection and experience exchange

Conversion of excisers	(i) Reduce availability of excisers (ii) Easy success indicators (iii) Media coverage providing visibility to issue	(i) Does not reduce demand for FGM (ii) Continue secretly or by apprentices (iii) Others take over task (iv) Ex-excisers unreliable sources against FGM (v) Alternative income may not motivate abandonment	(i) Ensure that work with excisers is only an aspect of a wider approach adapted to their roles in the particular community. (ii) Do not expect it to reduce the demand for FGM

Training of health professionals	(i) Improved quality of care (ii) Refrain to perform FGM (iii) Provide information and counselling (iv) Build local evidence on health consequences	(i) Resistance to work against FGM (ii) Inadequate content of training (iii) Lack of time and recourses to implement	(i) Comprehensive training for prevention and management into standard curricula (ii) Training target potential acceptance of the practice. (iii) Ensure an enabling environment for implementation of knowledge.

Alternative rites	(i) Facilitate community ownership and support, as it maintains key cultural practice (ii) Increased knowledge and empowerment of girls (iii) Publicity about change through community celebrations	(i) Only viable in communities in which FGM is a part of a rite of passage (ii) Limited integration of the whole community (iii) Insufficient adaptation into the specific sociocultural situation of each community	(i) Use only where fit into local culture (ii) Include the whole family and community (iii) Consider alternative measures if the actual cutting is done at other times (iv) Ensure community ownership for sustainability.

Community-led	(i) Community own problem and solution (ii) Broader support, less resistance (iii) Addressing underlying causes (iv) Reduce/remove FGM as a social norm, facilitating and stimulating change	(i) The community might decide to change, rather than to abandon, the practice. (ii) Failing to ensure community participation and resorting to traditional “lecturing”	(i) Ensure community ownership and adaptation (ii) Ensure long-term support to secure viable and broad change, reaching reluctant abandoners and neighbouring communities

Public statements	(i) Create a sense of social change among a group (ii) Facilitate and stimulate abandonment for group members	(i) Public statements by subgroups only lack of community ownership (ii) “Fake” opinions and/or lack of authority	(i) Ensure community-wide support (ii) Having legislation and policies in place provides support to people ready to change (iii) Further research is needed to investigate the effects of public statements from single groups, especially religious leaders (e.g., fatwas).

Legal measures	(i) Create an enabling framework (ii) Discourage FGM	(i) Practice can go underground (ii) Fear of seeking health care for complications	(i) Ensure community support for the law (ii) Ensure regulations that guarantee care for complications.

## References

[1] UNICEF (2005). *Female Genital Mutilation/Cutting. A Statistical Exploration*.

[2] UNFPA

[3] World Health Organisation Eliminating Female genital mutilation. An interagency statement.

[4] Yoder S, Wang S, Johansen REB Female genital mutilation/cutting: estimates of numbers from national surveys in 28 countries with national surveys.

[5] UNICEF (2009). Progress for children. *A Report Card on Child Protection*.

[6] Denison E, Berg R, Lewin S, Fretheim A (2009). Effectiveness of interventions designed to reduce the prevalence of female genital mutilation/cutting.

[7] Toubia NF, Sharief EH (2003). Female genital mutilation: have we made progress?. *International Journal of Gynecology and Obstetrics*.

[8] World Health Organisation (1999). *Female Genital Mutilation. Programmes to Date: What Works and What Doesn't?*.

[9] Berg R, Denison E (2012). Effectiveness of interventions designed to prevent female genital mutilation/cutting (FGM/C): a systematic review of the best available evidence. *Studies in Family Planning*.

[10] Diop N, Askew I, Abusharaf RM (2006). Strategies for encouraging the abandonment of female genital cutting: experiences from Senegal, Burkina Faso and Mali. *Female Circumcision*.

[11] Muteshi J, Sass J (2005). *Female Genital Mutilation in Africa: An Analysis of Current abAndonment Approaches*.

[12] Population Reference Bureau (2006). *AbAndoning Female Genital Mutilation/Cutting: An in-Depth Look at Promising Practices*.

[13] Lunde IB (2012). *Eradicating female genital cutting: understading reality conceptions. A study on perceptions of female genital cutting in Hargeisa, Somaliland (Somalia) [Ph.D. thesis]*.

[14] World Health Organisation Global strategy to stop health-care providers from performing female genital mutilation.

[15] UNICEF (2010). *The Dynamics of Social Change: Towards the Abandonment of FGM in Five African Countries*.

[16] Kaplan A, Forbes M, Bonhoure I (2011). Female genital mutilation/cutting (FGM/C) in The Gambia: long-term health consequences and complications during delivery and for the new born. *BMC Public Health*.

[17] UNFPA UNFPA-UNICEF Joint Programme on Female Genital Mutilation/Cutting: Accelarating Change.

[18] Barsoum B, Rifaat N, El-Gibaly O, Elwan N, Forcier N (2011). National efforts towards FGM-free villages in Egypt: the evidence of impact.

[19] Violence is not our culture. Mauritania: Muslim imams initiate rare ban on female circumcision.

[20] Latourès A (2008). *Saisir l'Etat en action en Afrique subsaharienne: action publique et appropriation de la cause des MGF au Mali et au Kenya [Ph.D. thesis]*.

[21] Kaplan A, Hechavarria S, Martin M, Bonhoure I (2011). Health consequences of female genital mutilation/cutting in the Gambia, evidence into action. *Reproductive Health*.

[22] Johansen REB (2011). Health professionals should never perform female genital mutilation. *Harmful Practices and Human Rights*.

[23] Johansen REB (2006). *Experiences and perceptions of pain, sexuality, and childbirth. A study of female genital cutting among Somalis in Norwegian exile, and their health care providers [Ph.D. thesis]*.

[24] Satti A, Elmusharaf S, Bedri H (2006). Prevalence and determinants of the practice of genital mutilation of girls in Khartoum, Sudan. *Annals of Tropical Paediatrics*.

[25] Hadi AA, Abusharaf RM (2006). A community of women empowered: the story of Deir Al Barsha. *Female Circumcision*.

[26] Shell-Duncan B, Hernlund Y, Wander K, Moreau A (2010). Contingency and change in the practice of female genital cutting: dynamics of decision making in Senegambia.

[27] Gosselin C (2000). Handing over the knife: Numu women and the campaign against women in Mali. *Transcultural Bodies: Female Genital Cutting in Global Context*.

[28] Oloo H, Wanjiru M, Newell-Jones K (2011). Female genital mutilation practices in Kenya: the role of alternative rites of passage. A case study of Kisii and Kuria districts. *Feed the Minds*.

[29] Mohamud M, Radeny S, Ringheim K, Abusharaf RM (2006). Community-based efforts to end female genital mutilation in Kenya: raising awareness and organising alternative rites of passage. *Female Circumcision*.

[30] Banks E, Meirik O, Farley T, Akande O, Bathija H, Ali M (2006). Female genital mutilation and obstetric outcome: WHO collaborative prospective study in six African countries. *The Lancet*.

[31] Yoder S, Khan S (2008). *Number of Women Circumcised in Africa: the Production of A Total*.

[32] Diop N, Sangaré M, Tandia F, Touré K (1998). *Etude de l'efficacité de la Formation du Personnel Socio-Sanitaire dans l'Éducation des Client(e)s sur l'Excision et dans le Traitement de ses Complications au Mali*.

[33] Hernlund Y External review of the training and information campaign on the eradication of FGM in the Gambia.

[34] Population Council and CNRST (1998). Evaluation de la stratégie de reconversion des exciseuses pour l'éradication des mutilations génitales féminines au Mali. *Rapport final du projet de recherche operationnelle et d'assistance technique en Afrique II*.

[35] GTZ (2001). *Addressing Female Genital Mutilation. Challenges and Perspectives For Health Programmes. Part I: Selected Approaches*.

[36] Population Council and USAID (2006). *Reproductive Health Update Trainings For Health Workers in North Eastern Province*.

[37] Refaat A (2009). Medicalization of female genital cutting in Egypt. *Eastern Mediterranean Health Journal*.

[38] Kaplan A, Hechavarria S, Bernal M, Bonhoure I Knowledge, attitude and practices regarding FGM/C among rural Gambian health care professionals: a transcultural study.

[39] Hadi AA, Salaam AS (1999). *Physicians Attitudes Towards Female Circumcision*.

[40] Njue C, Askew I (2004). *Medicalisation of Female Genital Cutting among the Abagusii in Nyanza Province, Kenya*.

[41] Rasheed SM, Abd-Ellah AH, Yousef FM (2011). Female genital mutilation in Upper Egypt in the new millennium. *International Journal of Gynecology and Obstetrics*.

[42] Mounir GM, Mahdy NH, Fatohy IM (2003). Impact of health education program about reproductive health on knowledge and attitude of female Alexandria University students. *The Journal of the Egyptian Public Health Association*.

[43] Berg R, Denison E, Fretheim A (2009). Factors promoting and hindering the practice of female genital mutilation/cutting (FGM/C). *Norwegian Knowledge Centre for the Health Services Report*.

[44] Hernlund Y, Shell-Duncan B, Hernlund Y (2000). Cutting without ritual and ritual without cutting: female, circumcision and the re-ritualisation of initiation in the Gambia. *Female, “Circumcision” in Africa: Culture, Controversy and Change*.

[45] Chege JN, Askew I, Liku J (2001). *An Assessment of the Alternative Rites Approach For Encouraging Abandonment of Female Genital Mutilation in Kenya*.

[46] Population Reference Bureau (2001). *Abandoning Female Genital Cutting. Prevalence, Attitudes and Efforts To End the Practice*.

[47] Dellenborg L (2009). From pain to virtue, clitoridectomy and other ordeals in the creation of a female person. *SIDA Studies*.

[48] Ahmadu F, Shell-Duncan B, Hernlund Y (2000). Rites and wrongs: an insider/outsider reflects on power and excision. *Female, “Circumcision” in Africa: Culture, Controversy and Change*.

[49] Mackie G, Shell-Duncan B, Hernlund Y (2000). Female genital cutting: the beginning of the end. *Female, “Circumcision” in Africa: Culture, Controversy and Change*.

[50] World Health Organisation (2012). *Community Empowerment, Internet Communication*.

[51] Berg RC, Denison E (2013). Interventions to reduce the prevalence of female genital mutilation/cutting in African countries. *Campbell Systematic Reviews*.

[52] UNICEF Somalia.

[53] Draege TL (2007). *The role of men in the maintenance and change of female genital cutting in Eritrea [Ph.D. thesis]*.

[54] Asmani IL, Abdi MS (2008). *De-Linking Female Genital Mutilation/Cutting From Islam*.

[55] Aberese Ako M, Akweongo P (2009). The limited effectiveness of legislation against female genital mutilation and the role of community beliefs in Upper East Region, Ghana. *Reproductive Health Matters*.

[56] Sipsma HL, Chen PG, Oforiatta A, Ilozumba UO, Karfo K, Bradley EH (2012). Female genital cutting: current practices and beliefs in western Africa. *Bulletin of the World Health Organization*.

[57] Diop N, Congo Z (2008). *Analysis of the Evoluation of the Practice of Female Genital Cutting in Burkina Faso*.

[58] Rogers EM (1962). *Diffusion of Innovations*.

